# The intra-articular use of ropivacaine for the control of post knee arthroscopy pain

**DOI:** 10.1186/1749-799X-1-17

**Published:** 2006-12-23

**Authors:** Efthimios P Samoladas, Byron Chalidis, Hlias Fotiadis, Ioanis Terzidis, Thomas Ntobas, Miltos Koimtzis

**Affiliations:** 1 Orthopaedic Department, Veria Hospital, Greece

## Abstract

**Aims:**

The purpose of this prospective randomised study is to evaluate the efficacy, safety and the appropriate dose of the ropivacaine in the control of post-knee arthroscopy pain.

**Methods:**

We randomised 60 patients in two groups to receive 10 ml/7.5 mg/ml ropivacaine (Group B) or 20 ml/7.5 mg/ml (Group A) at the end of a routine knee arthroscopy. We monitored the patient's blood pressure, heart rate, allergic reactions, headache, nausea, we assessed the pain using the visual analogue score at intervals of 1,2,3,4 and 6 hours after the operation. and we recorded the need for extra analgesia.

**Results:**

The intraarticular use of the ropivacaine provided excellent control of pain after knee arthroscopy. At two hours post-operatively there wasn't any difference between the two groups. Afterwards, the Group A showed increased pain and need for supplementary medication.

**Conclusion:**

We believe that intraarticular use of ropivacaine is effective to reduce post-operative pain minimising the use of systematic analgesia.

## Background

Arthroscopic knee surgery is one of the most common surgical procedures done in an outpatient setting. Post operative pain undoubtedly, it has a negative impact on the patient's psychology causing discomfort and prohibiting early mobilisation.

Administration of oral opioids and non-steroid antinflammatory drugs are combined with sufficient relief of pain in the immediate postoperative period [9]. However, they aren't site-specific and can be burdened by side effects, such as respiratory depression, nausea or acute gastric lesions, Early post operative pain following arhroscopic knee surgery is well controled with the use of a local anaesthetic agent. This has confirmed in many controlled studies [1,2].

Although the pain has been reported slight to moderate and of short duration, a review of 20 studies showed evidence for reduction in postoperative pain after intra-articular local anaesthesia following arthroscopic knee surgery [3]. No adverse effects or toxicity attributable to the intra-articular administration of local anaesthetics were reported in this review [4].

Ropivacaine is a new local amino-amide anesthetic that blocks peripheral afferents from acting on voltage-dependent Na^+ ^channels. It has the similar pharmacokinetic properties as bupivacaine but different pharmacodynamic such as their vasodilatory property and the toxicity. Furthermore, ropivacaine has a lower molecular weight than bupivacaine (Ropi 262 vs Bupi 288) The chance to use this drug in high concentrations provides higher clinical efficacy [5]. The purpose of this study was to compare the analgesic efficacy of ropivacaine 7,5 mg/ml in post knee arthroscopy pain. We test whether 20 ml of 7,5 mg/ml ropivacaine gives better analgesia than 10 ml of 7,5 mg/ml ropivacaine.

## Patients and methods

After the approval of local scientific committee and signed informed consent, 60 patients (45 males and 15 females) with mean age of 33 years (range 19–50 years) and ASA I-II(American Society of Anesthesiologists) physical status, were scheduled for a routine elective knee arthroscopy. Patients with a history of sensitivity to local anaesthetics or preoperative administration of opioids or any other analgesics in the preceding 48 h were excluded from the study. Surgical interventions were diagnostic arthroscopies, meniscal excision or repair, removal of loose bodies and arthroscopic debridement. Cases of extensive arthroscopic synovectomy, ligament reconstruction and articular cartilage procedures were excluded.

All the arthroscopies were performed under tourniquet application and by the same surgeon. Two standard portals (anteromedial and anterolateral) were used and the mean duration of the whole procedure was 45 min (range 35 to 60 min).

Standardised general anaesthesia was selected in all the cases. Muscle relaxants and short-acting opioids (fentanyl) were used at the beginning of the operation. No nonsteroidal anti-inflammatory drugs or additional pain medications were administered.

Patients were divided in two groups of 30 patients in each group using randomly sealed envelopes. Group B patients received ropivacaine 10 ml of 7,5 mg/ml and Group A patients received ropivacaine 20 ml of 7.5 mg/ml at the end of surgery.

At the postoperative period we record the heart rate, blood pressure, allergic reactions, nausea and headache for the first 6 hours. Visual Analogue Score (VAS) for pain scores was recorded at intervals of 1, 2, 3, 4 and 6 h after the intra-articular injection.

In case of need of supplementary analgesics, 1 g paracetamole plus codeine phosphate 60 mg was administered. In the event that there was no pain relief, 75 mg i.m diclophenac was injected. The time to the first request for analgesia and the total analgesic requirements were recorded.

The data were analysed using T-test for VAS and Chi-square test for analgesic consumption.

## Results

**Figure 1:**
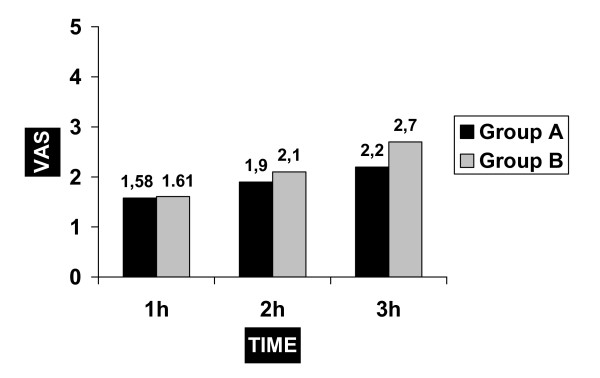
The comparison of VAS values of the groups at 1,2,3 hours.

No statistical difference regarding the VAS at the first 2 hours was detected between the two groups. At 3 hours post-operatively there was statistically lower VAS in Group A than in Group B (fig 1). After that time and if required, additional analgesia was admitted. At 4 and 6 hours post-operatively the VAS didn't show any difference but the result was affected by the potential use of supplementary analgesics.

As mentioned above none of the patients required extra analgesia until 3 hours post-operatively. Afterwards, Group B used significantly more analgesics than Group A. (p < 0,5).

No adverse reaction has been recorded between the groups.

## Discussion

Many modes of postoperative analgesia have been reported for patients undergoing knee arthroscopy [8]. The use of opioid drugs, administered by means of either patient-controlled analgesia or other methods, deals with postoperative pain efficiently but is often associated with side effects, including nausea and vomiting, respiratory depression, drowsiness, pruritus, reduced gut motility, and urinary retention [7]. Providing analgesia locally is an attractive option with minimal systemic side effects, and may lead to an earlier discharge from the hospital. Intraarticular drug administration is one of the simplest techniques requiring no specialised equipment for pain management after arthroscopic knee surgery [11].

Ropivacaine is a commonly used local anaesthetic and it is related structurally to bupivacaine and mepivacaine. It is a less lipid soluble than bupivacaine, but its pharmacokinetic disposition is similar. Ropivacaine seemed to provide similar and effective post-arthroscopy analgesia [6] compared to bupivacaine, showing less Central Nervous System (CNS) and cardiac toxicity [6].

Plasma concentrations of ropivacaine has been studied by Convery et al [4] and they found that for all patients and all doses (100–200 mg) fell below the estimated toxic thresholds, and therefore it seems that ropivacaine can be safely administered by intra-articular injection. Furthermore, Francesco et al reported that intra-articular administration of ropivacaine is as effective as morphine in controlling pain during the first 24 hours after knee arthroscopy, but it has an earlier onset than morphine [7].

The acute and most serious adverse effects of local anesthetics involve the CNS and the cardiovascular system. They usually occur either because of accidental intravascular or intrathecal injections, or a pronounced overdose. CNS symptoms of local anesthetic toxicity occur before cardiovascular symptoms and signs, and include numbness of the tongue, light-headedness, visual disturbances, and muscular twitching; more serious signs include seizures, coma, respiratory arrest, and cardiovascular depression. Extremely high concentrations depress spontaneous pacemaker activity in the sinus node and result in sinus bradycardia and sinus arrest. In our study none of the patients developed any adverse reactions therefore we assume that intraarticular dose of ropivacaine up to 150 mg is safe.

Our results revealed that there is no difference in pain between 10 ml and 20 ml of 7,5 mg/ml, at 2 hours postoperatively. At 3 hours there was a statistical difference in VAS and this was also confirmed by the use of supplementary analgesia.

After arthroscopy, acute inflammation is induced by the release of mediators from damaged cells. Martin et al noted that cryotherapy works in the acute inflammatory response decreasing the narcotic consumption, pain and knee swelling [10]. Although we didn't use cold therapy in our cases, we believe that the promising results of cryocuff application necessitates its use after knee arthroscopy procedures.

As a conclusion, intraarticular injections of local anaesthetics seems to provide an alternative and effective solution in pain control after knee arthroscopy.

**Table 1:** Group A Number of patients and VAS

**VAS**	**1H**	**2H**	**3H**	**4H**	**6H**
1	18	17	15	13	11
2	7	7	7	6	6
3	5	6	5	6	6
4			3	3	4
5				1	3
6					
7					
8				1	
9					
10					

**Table 2:** Group B Number of patients and VAS

**VAS**	**1H**	**2H**	**3H**	**4H**	**6H**
1	17	13	11	12	12
2	7	6	5	6	5
3	6	6	5	5	5
4		3	4	5	4
5		1	3	2	4
6					
7			1		
8		1	1		

**Table 3:** Number of patients requiring extra analgesia

	**EXTRA DRUG 3RD**	**EXTRA DRUG 4TH**	**EXTRA DRUG 6TH**	**EXTRA DRUG OVERAL**
**GROUP A**	3	6	9	18
**GROUP B**	10	7	8	25
